# Fetal heart and surroundings: umbilical cord traction triggers sympathetic heart rate surge in fetal lambs in the artificial womb

**DOI:** 10.3389/fmed.2025.1667607

**Published:** 2025-11-04

**Authors:** Maria Antonietta Castaldi, Salvatore Giovanni Castaldi, Maria Silena Mosquera, Grace Hwang, Marina Heffelfinger, Aaron Weilerstein, Marcus G. Davey, Alan W. Flake

**Affiliations:** ^1^Center for Fetal Research, Children’s Hospital of Philadelphia, Abramson Research Center, Philadelphia, PA, United States; ^2^Department of Medicine and Surgery, University of Salerno, Fisciano, Italy; ^3^Westchester Medical Center, Valhalla, NY, United States; ^4^Vitara Biomedical Inc., Philadelphia, PA, United States

**Keywords:** fetal heart rate, tachycardia, cardiotocography, artificial womb, mechanical activation, sheep (ovine), sympathetic nervous system

## Abstract

**Objective:**

To investigate whether umbilical cord traction triggers a unique umbilico-neuro-cardio reflex via sympathetic autonomic nervous system (ANS) activation, potentially explaining fetal heart rate (FHR) increases such as the double mountain peak sign and unexplained tachycardia outside labor.

**Design:**

Experimental physiological study using the EXTrauterine Environment for Neonatal Development (EXTEND) system.

**Setting:**

Controlled laboratory environment at The Children’s Hospital of Philadelphia with fetal lambs supported by the EXTEND system.

**Population or sample:**

Twenty-nine experiments were performed on three fetal lambs: 5 ANS-immature (<110 days gestational age) and 24 ANS-mature (>110 days GA), including subgroups for pharmacological intervention.

**Methods:**

Umbilical cord traction was performed in 20 experiments (5 ANS-immature, 15 ANS-mature). To investigate autonomic modulation, nine ANS-mature experiments included administration of either periumbilical capsaicin or intravenous propranolol. FHR changes were measured, and norepinephrine levels were sampled before and after interventions.

**Main outcome measures:**

Change in FHR (bpm) and circulating norepinephrine concentrations in response to umbilical cord traction and pharmacologic modulation.

**Results:**

Cord traction significantly increased FHR in ANS-mature lambs (mean +42 bpm, *p* < 0.001), but not in ANS-immature lambs (mean +1 bpm, *p* = 0.770). Capsaicin also elevated FHR and norepinephrine, while propranolol inhibited both responses.

**Conclusion:**

Umbilical cord traction appears to activate an umbilico-neuro-cardiac reflex through sympathetic ANS pathways in ANS-mature fetuses. This mechanism may underlie clinical CTG features such as the double mountain peak sign and fetal tachycardia outside of labor.

## 1 Introduction

Cardiotocography (CTG) is currently the mainstay of intrapartum fetal heart rate (FHR) monitoring worldwide (1). Several guidelines have been developed to interpret CTG findings and prevent fetal hypoxia/acidemia; however, international consensus is lacking (2). Moreover, the retrospective application of artificial intelligence or machine learning to CTG interpretation, while promising, has not been associated with measurable improved neonatal outcomes in terms of neonatal acidosis, cord blood pH <7.15–7.20, a 5-min Apgar score <7, mode of delivery, neonatal intensive care unit admission, neonatal seizures, or perinatal death (3–5).

Modern interpretations of CTG findings are primarily based on cardiovascular physiology (6). The goal is to identify whether the fetus is unable to adequately compensate for ongoing hypoxic and/or mechanical stress based on the features observed on the CTG trace (7). Failure to understand fetal physiology during labor and correlate the CTG patterns with the clinical picture may result in an increase in unnecessary operative interventions and/or an increase in the risk of intrapartum hypoxic injury leading to hypoxic-ischemic encephalopathy, severe metabolic acidosis culminating in long-term neurological sequelae (cerebral palsy), or perinatal death (6–8). In this cardiophysiological approach, fetal bradycardia (deceleration) is interpreted as a cardiac reaction to hypoxia.

Nevertheless, in clinical practice, there are several circumstances in which interpretation of the intrapartum FHR pattern falls outside the usual framework of reference [8]. The unanswered question is whether the current CTG interpretation effectively prevents both fetal hypoxia and adverse neonatal outcomes (9, 10). Thus, when considering CTG, not only the cardiovascular response to hypoxia/acidemia but also the physiological response to mechanical stimuli and biomechanical phenomena should require consideration. For instance, external cephalic version from a podalic presentation is often complicated by severe FHR abnormalities, mainly an abnormal acceleration or a tachycardia (2, 7, 10, 11).

Our hypothesis for this study was that an increase in FHR is triggered by a mechanical stimulus, specifically stretching of the umbilical cord. Such stimulation may occur spontaneously, as in a podalic version or intrauterine repositioning, or during clinical maneuvers such as an external cephalic version. Similar mechanical triggers may also arise in the presence of a very short cord. In addition, fetal exposure to nociceptive stimuli in a clinical setting could likewise elicit an increase in FHR, most notably during intrauterine interventions such as fetal surgery, intrauterine transfusion, or shunt placement; during complicated labor involving forceful manipulations; or in association with fetal conditions (e.g., intrauterine growth restriction or infection), where hypoxia and inflammation can sensitize nociceptive pathways. Importantly, these clinical contexts often combine mechanical and nociceptive components (i.e., excessive traction can be painful), which may act synergistically to modulate FHR. Additionally, we propose that the resulting FHR acceleration (7) or tachycardia (11) constitutes an epiphenomenon that emerges only in moderate-to-late preterm and term pregnancies (12). Indeed, from 32 weeks’ gestation onward, physiological control of the FHR and the resultant features observed on the CTG trace do not significantly differ in moderate-to-late preterm versus term fetuses (13) and depend on the activity of an almost fully developed sympathetic nervous system (SNS) (14).

To test this hypothesis, we used the EXTrauterine Environment for Neonatal Development (EXTEND) system. Previous data demonstrated that lambs on the EXTEND system maintain normal fetal physiology, circulation, organ development, normal growth, and maturation at different gestational ages (GA), ranging from 90–95 days of GA (15), to 105–117 days of GA (16). Using this system, which provides direct access to the umbilical cord of fetal lambs, we assessed the fetal physiological response to cord stretching independently of maternal influences and pharmacological effects (15, 16).

## 2 Materials and methods

### 2.1 EXTEND model

Experiments were performed on lambs supported by the EXTEND system in accordance with the protocols approved by the Institutional Animal Care and Use Committee of The Children’s Hospital of Philadelphia (IAC 22-000984_AM12), as part of a larger study designed to evaluate long-term fetal survival under conditions closely replicating the physiological environment of the maternal womb. The study was designed to allow direct access to the umbilical cord under quasi-physiological conditions (15, 16). Indeed, no other experimental model currently enables direct access to the fetus and umbilical cord while simultaneously providing sustained life support, as the EXTEND system does. This system has demonstrated the capacity to support fetal lambs for up to 28 days without the use of analgesia or anesthesia (15, 16). In this study, three fetal lambs delivered prematurely (at 91-, 92-, and 93-days’ GA) via hysterotomy were transitioned to a sterile fluid environment where they were supported within the EXTEND system (15, 16). The extracorporeal circuit consisted of minimal lengths of tubing and a low-resistance low-volume oxygenator primed with maternal blood. Umbilical vessel cannulation was achieved avoiding any manipulation of the fetal portion of the umbilical cord. The runs on the EXTEND system ended at 113-, 116-, and 109-days’ GA, lasting, respectively, 22, 16 and 24 days. The animal characteristics are presented in Supplementary Table 1. It is important to note that analgesia and anesthesia were administered solely to the pregnant ewe at the time of surgery, specifically for performing the cesarean section required to place the fetal lambs on the EXTEND system.

The timing of our studies based on ANS maturation in sheep. Fetal lamb ANS activity, though not fully mature, has been showed to occur around 110 days GA (17–19), which can be compared to human pregnancies from 32 weeks’ onward (14, 20). Thus, we performed experiments on animals that were termed as ANS-immature, and ANS-mature lambs, considered as before and after 110 days GA, respectively.

Physiological monitoring during EXTEND was performed as previously described (15, 16). Monitored hemodynamic parameters in these fetal lambs included FHR (reported in beat per minute, bpm), mean pre-membrane circuit pressure (MACP), calculated as 1/3 systolic + 2/3 diastolic pressure and representing the pre-membrane circuit pressure measured on the pre-oxygenator limb of the circuit. This measurement reflects the resistance imposed by the circuit and oxygenator on venous return from the fetus. Values are reported as mmHg. Mean circuit blood flow (FLOW) was continuously measured using ultrasonic transit-time flow probes placed on the umbilical-vein limb of the circuit (post-oxygenator). Values are reported as mL/min. Because the system is pumpless, flow is generated entirely by the fetal heart and reflects circuit resistance, including the cannulae and oxygenator (15, 16).

Oxygen delivery (DO₂) was calculated as blood flow multiplied by arterial oxygen content (CaO₂), with CaO₂ derived from hemoglobin concentration, oxygen saturation, and partial pressure of oxygen according to standard neonatal equations. Blood gases were sampled via catheter ports positioned on both the arterial and venous limbs of the circuit (15, 16). This approach ensured that oxygen delivery could be directly compared with physiological fetal ranges and monitored continuously throughout the experiments. Moreover, for the purposes of the present study, DO₂ was selected over blood gas analysis due to its capacity for rapid change, making it an ideal parameter for real-time evaluation. Data were continuously measured and recorded on LabChart 8 (AD Instruments Inc., Colorado Springs, CO, United States).

### 2.2 Experimental design

This study was conceived by one author (MC) after the clinical observations of two different events manifest by abnormal acceleration and fetal tachycardia that were presumably linked to mechanical traction of the umbilical cord and/or nociceptive stimulus. Specifically, the abnormal onset of FHR acceleration during uterine contractions in advanced labor in a case of absolute brevity of the umbilical cord in a 41-weeks’ gestation pregnancy, and the onset of a severe tachycardia after a spontaneous cephalic version in a 37-week gestation pregnancy.

**Figure 1:**
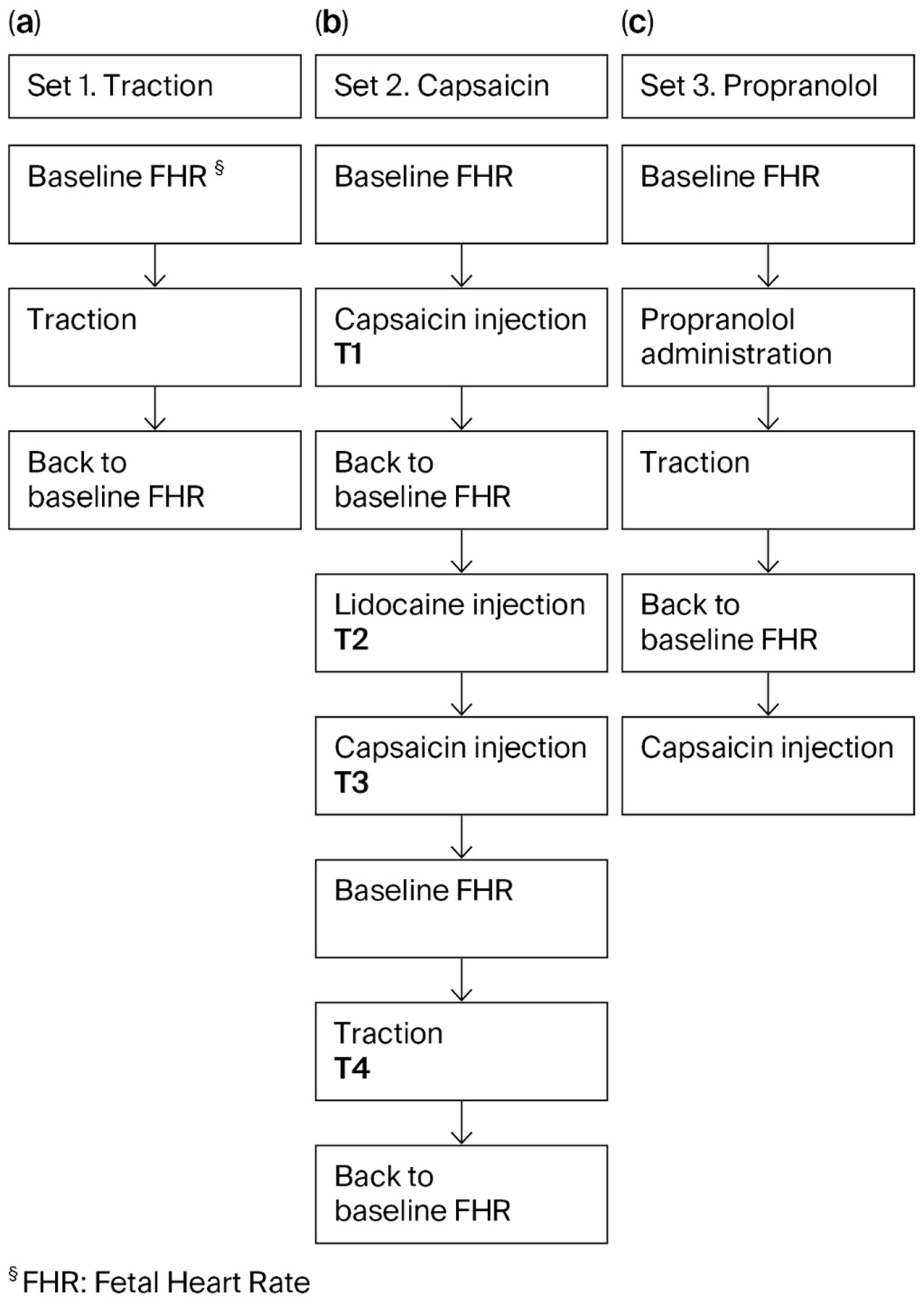
Schematic representation of the protocols involved in the study. Time course of the steps, together with mechanical and pharmacological manipulations are shown. A total of 29 experiments were performed. **(a)** Set 1 traction: Twenty experiments were performed: 5 in ANS-immature* sheep (animal ID 7780L2 at 102-day), and 15 in ANS-mature* sheep (10 in animal ID 2995L1 at 112- and 113-days, and 5 in animal ID 7954L1 at 112-day). **(b)** Set 2 capsaicin: Four experiments were performed, T1–T4 (animal ID 7954L1 at 113-day). **(c)** Set 3 propranol: Five experiments were performed (animal ID 7954L1 at 113-day).

The experimental design consisted of three sets of experiments each of them involving a direct access to the umbilical cord, for traction or periumbilical injection of capsaicin, a pain-inducing compound (Figure 1); set 1 traction—umbilical cord traction was performed to determine if umbilical cord traction could mechanically trigger an increase in FHR as a protective response in ANS-mature and immature lamb fetuses; set 2 capsaicin—once traction was demonstrated to elicit an FHR acceleration in ANS-mature sheep, capsaicin was injected subcutaneously around the umbilicus to determine whether direct chemical stimulation of sensory C-fibers via periumbilical administration induces tachycardia. Subsequently, lidocaine was administered to block both sensory fiber activity and adrenergic signaling, in order to determine whether a second capsaicin injection or cord traction could still provoke a rise in FHR; set 3 propranolol—intravenous propranolol was administered prior to cord traction to assess the possible role of β-adrenergic signaling in this umbilico-neuro-cardiac response.

Blood plasma samples were collected at different times to measure norepinephrine concentrations, as explained in detail below, and analyzed using an enzyme-linked immunosorbent assay (ab287789—Norepinephrine ELISA Kit; Abcam Limited, Cambridge, CB2 0AX, United Kingdom).

We performed a total of 29 experiments. Each experiment is depicted in paired fashion with the timing of interventions shown in sequential order. Investigators were not blinded to the experimental conditions.

#### 2.2.1 Traction

**Figure 2:**
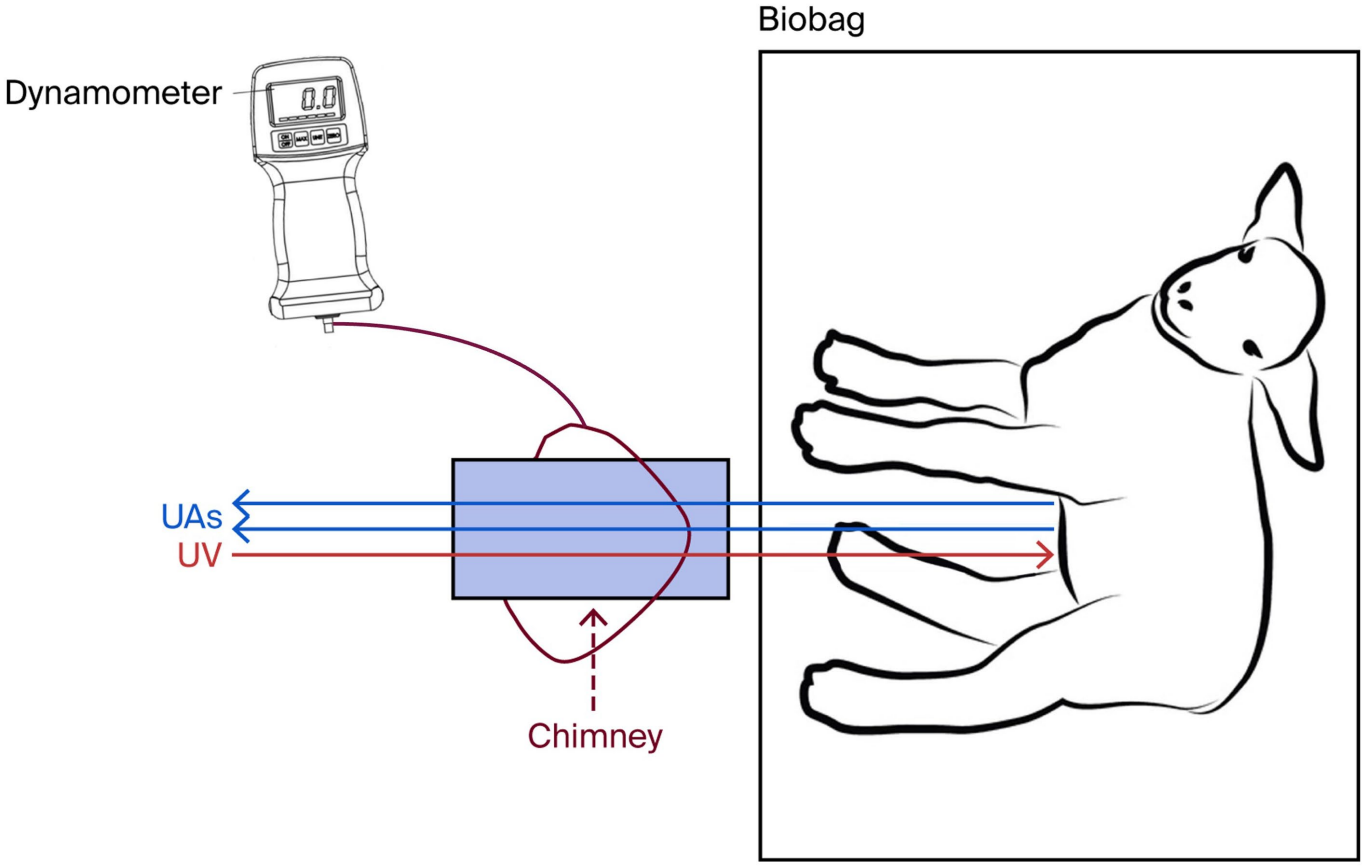
Controlled traction experiment in the EXTEND model. Controlled traction was obtained by using a dynamometer positioned around the plastic chimney containing the connections (burgundy dashed arrow) with the umbilical vessels. Then the lamb in the biobag was gently pushed toward the back wall, while the chimney was gently stretched in the opposite direction. (UAs, umbilical arteries; UV, umbilical vein).

Controlled traction of the umbilical cord was performed to obtain an increase of at least 20 bpm in FHR triggered by traction of the umbilical cord. Twenty experiments were performed in three different animals, divided into four subsets, each comprising five tractions (Figure 1a: set 1), imitating the physiology of normal labor (2, 21–24). Five experiments were performed in ANS-immature sheep (animal ID 7780L2 at 102-days’ GA, day 9 on circuit) versus 15 experiments in ANS-mature sheep [10 (animal ID 2995L1) at 112 days and 113 days GA, day 20 and 21 on circuit, five (animal ID 7954L1) at 112 days’ GA, day 21 on circuit]. A dynamometer was positioned around the plastic ‘chimney’—a structure housing the connections to the umbilical vessels—to monitor the applied tensile force. Once in place, the stabilizing supports of the chimney were removed, and the lamb was gently pushed toward the back wall of the biobag while the chimney was simultaneously stretched in the opposite direction for 45–90 s (mean duration: 63 s), without exceeding 1 N (25–27). This procedure was designed to mimic the duration and mechanical stress of uterine contractions during labor (Figure 2) (2, 21–24). The traction was interrupted when a response was elicited or 90 s passed. The mean interval between the experiments in each subset was 5:55 min. FHR, MACP, FLOW, and DO_2_ were continuously measured and recorded on LabChart 8 (AD Instruments Inc., Colorado Springs, CO, United States) and analyzed at the beginning of each experiment as well as at the peak of FHR during the experiment.

Blood plasma samples were collected at the beginning and end of each of the four experimental subsets to measure norepinephrine concentrations.

#### 2.2.2 Capsaicin

Upon confirmation of the presence of a significant increase in FHR following cord stretching in an ANS-mature animal, the second set of experiments (four experiments on animal ID 7954L1 at 113 days GA, day 22 on circuit, Figure 1b: set 2) aimed to verify the involvement of sympathetic activity in triggering tachycardia. A periumbilical subcutaneous injection of Capsaicin 25 mg/kg (12.5 mg/kg per side) (28) was administered after the biobag was opened (Figure 1b: set 2, T1). Lidocaine (1 mL of 1% in saline) was administered via a subcutaneous periumbilical injection 15 min after capsaicin administration to block the sensory nerves of the skin (29) (Figure 1b: set 2, T2). After a further 5-min interval, once FHR had returned to baseline, a second dose of capsaicin was administered (Figure 1b: set 2, T3). Finally, a traction experiment was performed as described above for set 1 (Figure 1b: set 2, T4). To measure norepinephrine concentrations, plasma samples were collected 5 min before and after T1 and T3.

#### 2.2.3 Propranolol

The third set (PROPRANOLOL) aimed to assess the role of β-adrenergic signaling in umbilico-neuro-cardiac reflex in an ANS-mature animal (five experiments performed on animal ID 7954L1 at 113 days GA, day 22 on circuit, Figure 1c: set 3). First, propranolol (2 mg/kg, Karnodyl; Primius Lab Ltd., London, United Kingdom) (30) was intravascularly administered into the circuit. Ten minutes later, three traction experiments were performed as described above for set 1, and a dose of capsaicin was administered after an interval of 5 min. To measure the norepinephrine levels, blood samples were collected 5 min after the three traction experiments and capsaicin injection.

### 2.3 Statistics

Power calculations were performed to determine the appropriate sample size for this study. Based on previous literature (31–35) and data from the EXTEND system (36), the mean FHR baseline was set at 176 bpm (± 6.7 bpm, 95% confidence interval 162–189 bpm), and a one-sample two-sided test power calculation was performed. This power calculation indicated that one animal would be necessary to detect a 15% increase in bpm (20 bpm) in FHR, with a power of 80% at a 0.5% significance level. Fetal heart rate (FHR) in ANS-mature vs. ANS immature lambs is the primary outcome variable under analysis. Traction experiments were conducted repeatedly in the same animal, a design deemed appropriate for addressing the objectives of the study.

Statistical analyses were performed using the SPSS version 29 (SPSS Inc., Chicago, IL, United States) and Microsoft Office 365 (2023; Microsoft Corporation, Redmond, WA, United States).

Shapiro–Wilk test of normality and Kolmogorov–Smirnov test for distribution assessment were performed and showed an overall parametric distribution. To analyze the differences in the considered parameters, a two-sided paired *t*-test sample analysis (*t*) and repeated measures ANOVA test (*F*) were performed depending on the normal distribution. Statistical significance was set at *p* < 0.05. Data are presented as mean ± standard error of mean, unless otherwise specified. Due to the small sample size, single measurement parameters from the second and third sets of the experiments are presented descriptively and graphically.

## 3 Results

### 3.1 Traction

**Figure 3:**
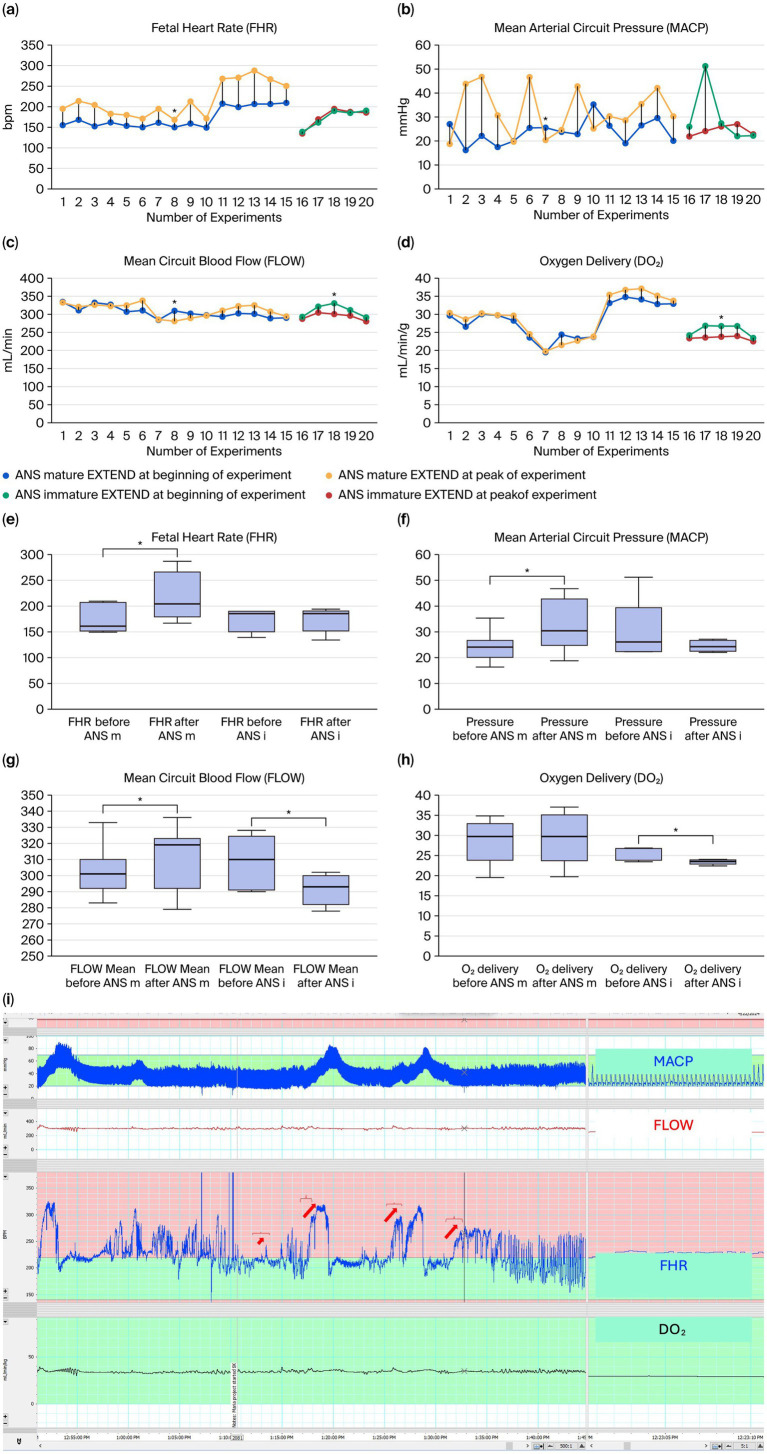
**(a–d)** Fetal heart rate (FHR), mean arterial circuit pressure (MCAP), mean circuit blood flow (FLOW) and oxygen delivery (DO_2_) variations in the 20 traction experiments performed. Statistical significance is expressed by * (*p* < 0.05). **(e–h)** Box plots of fetal responses to umbilical cord traction in ANS-mature and ANS-immature lambs. Panels show **(e)** fetal heart rate (FHR), **(f)** mean arterial circuit pressure (MACP), **(g)** mean circuit blood flow (FLOW), and **(h)** oxygen delivery (DO₂) at baseline and at the peak of traction. ANS-mature lambs (ANS m) displayed significant increases in FHR and MACP, whereas ANS-immature lambs (ANS i) showed significant decreases in FLOW and DO₂. Data are presented as mean ± SEM with 95% CI. Statistical significance is indicated by * (*p* < 0.05). **(i)** Graphic depiction of cord traction experiments visualized on LabChart 8 (AD Instruments Inc., Colorado Springs, CO, United States) with variations of FHR, MACP, FLOW and DO_2_ in the EXTEND system during a cord traction experiment in a 112-day lamb (animal ID 7954L1, day 21 of the run). The red symbol (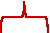) represents traction, and the red arrows the significant increases in FHR after 1-min tractions.

Our main goal was to determine whether an umbilico-neuro-cardiac reflex exists in fetal lambs and is caused by a sympathetic surge triggered by controlled traction. In ANS-mature lambs, cord traction elicited a significant increase in FHR, rising from a baseline of 172 ± 6 bpm (95% CI 149–209) to 215 ± 10 bpm (95% CI 167–287, *p* < 0.001). Mean arterial circuit pressure (MACP) also increased significantly, from 24.0 ± 1.3 mmHg (95% CI 16.4–35.4) to 32.5 ± 2.6 mmHg (95% CI 18.8–46.8, *p* = 0.007 (*t*); *p* = 0.14 ANOVA). No significant differences were observed for circuit blood flow (FLOW: 304 ± 3.9 vs. 309 ± 4.7 mL/min, *p* = 0.216) or oxygen delivery (DO₂: 28.4 ± 1.2 vs. 29.2 ± 1.5 mL/min/kg, *p* = 0.059) (Table 1 and Figures 3a–i).

**Table 1:** Mean fetal heart rate (FHR), mean arterial circuit pressure (MACP), mean circuit blood flow (FLOW) and oxygen delivery (DO_2_) at the beginning and at peak of the umbilical cord traction experiments in ANS^a^-mature and ANS-immature^b^ lambs on the EXTEND system^c^.

Variable	ANS-mature: beginning	ANS-mature: peak FHR	*t*-test		ANOVA	
			(*t*)	(*p*)	(*t*)	(*p*)
FHR (bpm)	172 ± 6 [149–209]	215 ± 10 [167–287]	−8.252	<0.001	47.963	<0.001
MACP (mmHg)	24.0 ± 1.3 [16.4–35.4]	32.5 ± 2.6 [18.8–46.8]	−2.797	0.007	7.825	0.014
FLOW (mL/min)	304 ± 3.9 [283–333]	309 ± 4.7 [279–336]	−1.295	0.216	1.677	0.216
DO₂ (mL/min/kg)	28.4 ± 1.2 [19.5–34.8]	29.2 ± 1.5 [19.7–37.0]	−2.056	0.059	4.225	0.059

Variable	ANS-immature: beginning	ANS-immature: peak FHR	*t*-test		ANOVA	
			(*t*)	(*p*)	(*t*)	(*p*)
FHR (bpm)	173 ± 10 [139–190]	174 ± 11 [134–194]	−0.313	0.770	0.098	0.770
MACP (mmHg)	29.9 ± 5.4 [22.2–51.2]	24.5 ± 1.0 [22.0–27.2]	0.960	0.392	0.921	0.392
FLOW (mL/min)	308 ± 8 [290–328]	291 ± 4 [278–302]	4.208	0.014	17.706	0.014
DO₂ (mL/min/kg)	25.6 ± 0.7 [23.4–26.8]	23.4 ± 0.3 [22.4–24.0]	4.264	0.013	18.181	0.013

a ANS, autonomic nervous system.

b ANS-immature vs. ANS-mature lambs were defined as before or after 110 days’ GA.

c Data are presented as mean ± S.E.M., and confidence interval 95%. Significance was set at *p* < 0.05.

In contrast, in ANS-immature lambs, cord traction did not significantly alter FHR (173 ± 10 vs. 174 ± 11 bpm, *p* = 0.770) or MACP (29.9 ± 5.4 vs. 24.5 ± 1.0 mmHg, *p* = 0.392). However, a significant decrease was observed in FLOW (308 ± 8 vs. 291 ± 4 mL/min, *p* = 0.014) and DO₂ (25.6 ± 0.7 vs. 23.4 ± 0.3 mL/min/kg, *p* = 0.013) (Table 1 and Figures 3a–h).

An upward trend in norepinephrine levels was observed in ANS-mature lambs (mean 1,191 ± 21 vs. 1,400 ± 252 pg/mL, *p* = ns), whereas no increase was observed in the ANS-immature lamb (1,445 vs. 1,315 pg/mL).

### 3.2 Capsaicin

**Figure 4:**
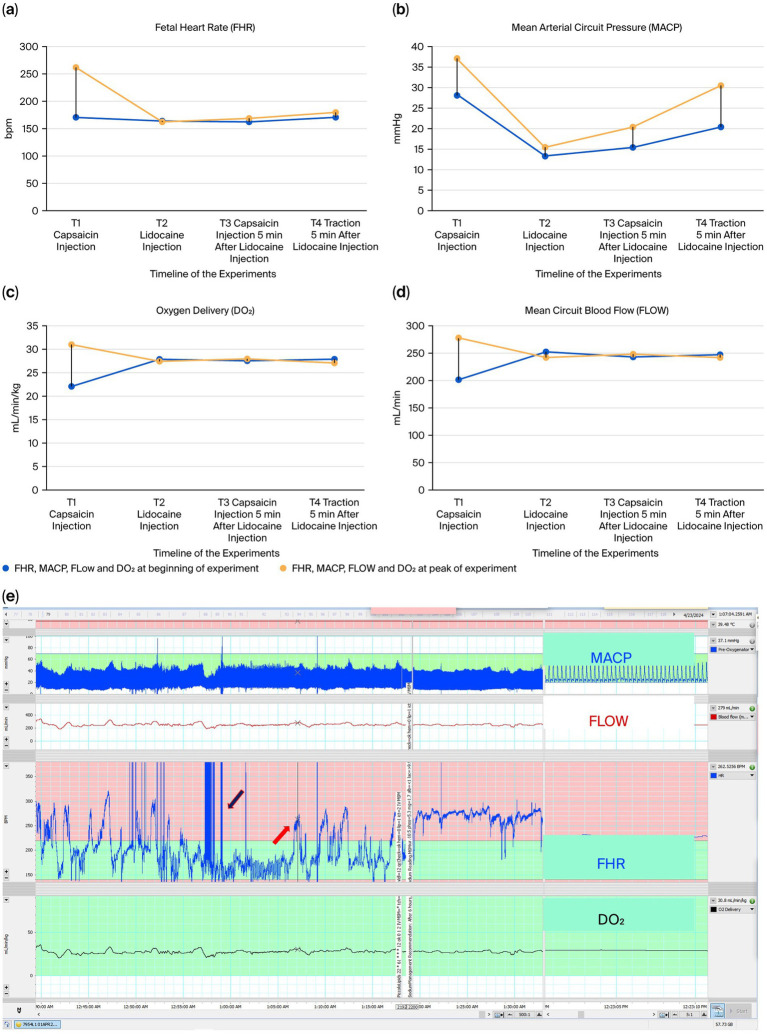
**(a–d)** Fetal heart rate (FHR), mean arterial circuit pressure (MCAP), mean circuit blood flow (FLOW) and oxygen delivery (DO_2_) variations in the capsaicin experiment set in ANS-mature lamb. **(e)** Graphic depiction of periumbilical subcutaneous injection of capsaicin 25 mg/kg (12.5 mg/kg per side) administered, after the biobag was opened (blue arrow) in a 113-day lamb (animal ID 7954L1, day 22 of the run). Ten minutes later a rise in FHR (red arrow) from 171 bpm to 268 bpm was observed. (LabChart 8AD Instruments Inc., Colorado Springs, CO, United States).

Second, to identify the importance of the ANS in triggering the umbilico-neuro-cardiac reflex, we directly stimulated the afferent C-fiber units around the umbilicus with capsaicin (29, 37). When capsaicin was injected subcutaneously around the umbilicus, a significant increase in fetal heart rate (FHR) was observed approximately 10 min later, consistent with the known onset time of subcutaneous capsaicin (10 min) (38), rising from 171 to 268 bpm (Figures 4a–e, T1). Moreover, norepinephrine levels increased after the capsaicin injection (1,371 vs. 1,674 pg/mL). No increase in FHR, MACP, FLOW, or DO_2_ was observed after the subcutaneous lidocaine injection (Figures 4a–d, T2). No increases in FHR, MACP, FLOW, or DO_2_ were observed after the capsaicin injection 5 min after the subcutaneous lidocaine injection blocked the sensory nerves (Figures 4a–d, T3). Finally, no increases in FHR, MACP, FLOW, or DO_2_ were observed after the traction experiment (Figures 4a–d, T4).

### 3.3 Propranolol

**Figure 5:**
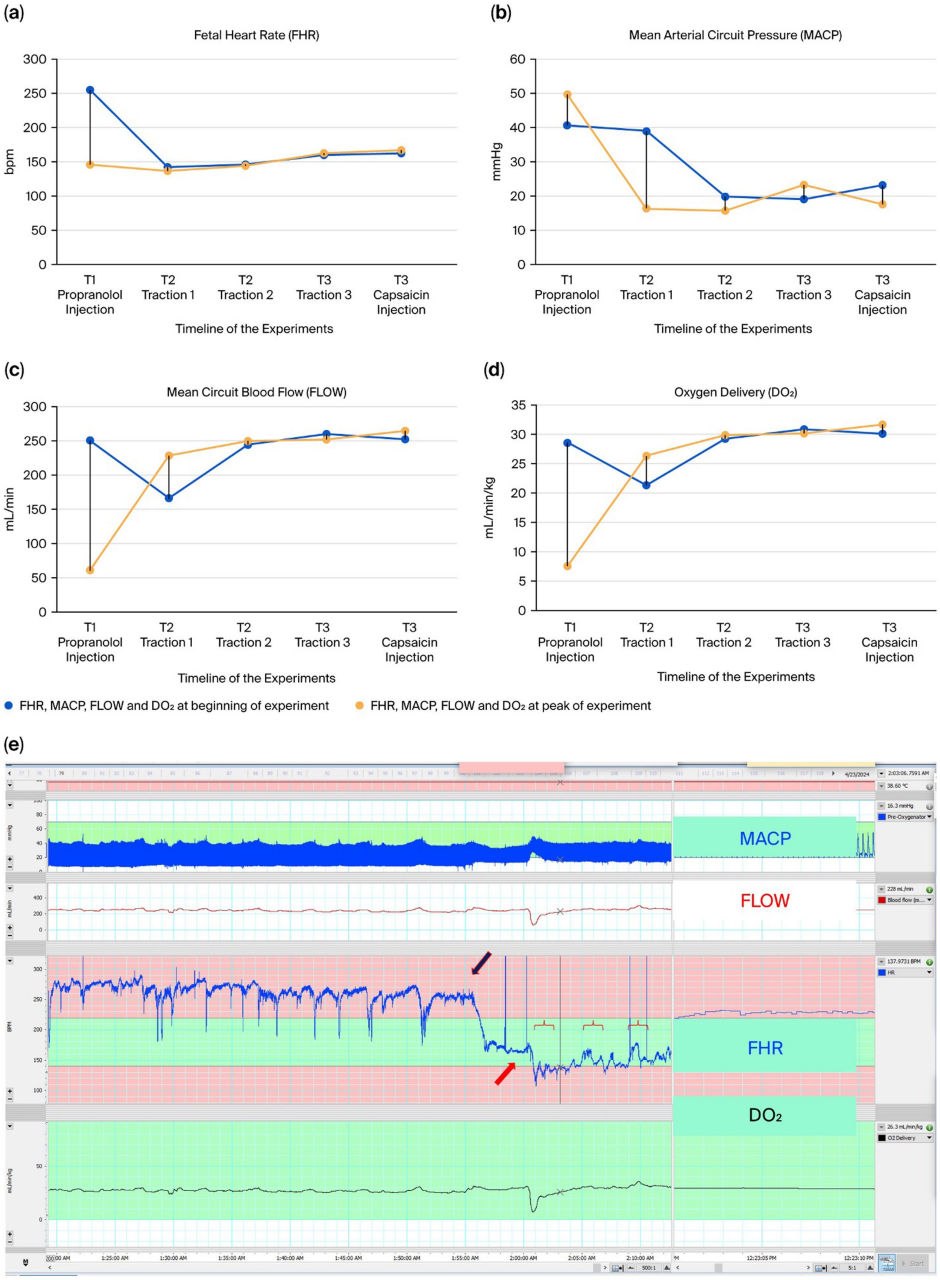
**(a–d)** Fetal heart rate (FHR), mean arterial circuit pressure (MCAP), mean circuit blood flow (FLOW) and oxygen delivery (DO_2_) variations in the propranolol experiment set in ANS-mature lamb. **(e)** Graphic depiction of intravenous propranolol (2 mg/kg) administration. A sudden drop in FHR from 255 (blue arrow) to 145 bpm (red arrow) was observed, and no raise in FHR was observed after 3 controlled tractions with a median of 143 bpm (red symbol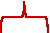). (LabChart 8 AD Instruments Inc., Colorado Springs, CO, United States).

Third, we investigated whether the administration of beta-blockers (propranolol), followed by either cord traction or capsaicin administration, would elicit a rise in FHR. When propranolol (2 mg/kg IV) was administered, a sudden drop in FHR from 255 to 145 bpm was observed. No increase in FHR was observed after the three controlled tractions, with a median of 143 bpm (range 137–162 bpm) (Figures 5a–e). A similar decrease was observed in MACP, FLOW, and DO_2_ after the capsaicin injection but not after the traction experiments (Figures 5a–e). Finally, no significant increase in norepinephrine levels was observed after propranolol administration (1,011 pg/mL) or the three controlled tractions (1,112 pg/mL).

## 4 Discussion

Our results, obtained in fetal lambs at different ANS developmental stages supported by the EXTEND system, showed that cord traction elicited a significant increase in FHR in ANS-mature lambs, together with MACP, whereas no significant increase was achieved in ANS-immature fetal lambs (Figures 3a–i and Table 1). In contrast, while no increase in FHR or MACP was observed in ANS-immature fetal sheep, a significant decrease in FLOW and DO_2_ was observed (Figures 3a–i and Table 1).

Moreover, we observed a consistent increase in norepinephrine levels in ANS-mature lambs versus almost no increase at earlier neurodevelopmental stages. Therefore, our hypothesis that cord traction could elicit fetal tachycardia via SNS activation was supported by our results. In fact, stretching of the cord of the ANS-immature animal leads to the expected results according to fluid mechanics, namely a reduction in the diameter of cord vessels that causes a reduction in blood flow. Additionally, DO_2_ is also reduced because it is a function of circuit flow, which is necessarily reduced without a contemporaneous increase in FHR or MACP (39). In contrast, in ANS-mature animals, the increase in FHR and MACP balances the fetal cord narrowing; thus, maintaining circuit flow and DO_2_.

Additionally, FHR elevation can also be triggered by skin nociception, as shown when capsaicin is injected subcutaneously around the umbilicus (Figures 4a–e). When lidocaine is administered, subsequent cord traction and capsaicin injection fail to produce any rise in FHR (Figures 4a–d). This is consistent with lidocaine’s dual action in suppressing nociceptive signaling and reducing adrenergic activation (29, 40–43). Moreover, the observed drop in baseline MACP following capsaicin infusion is within the expected range, and the apparent stability of circuit blood flow reflects the opposite-correlated behavior of flow and pressure, which is consistent with fluid mechanics. Eventually, when the adrenergic receptor was blocked by propranolol (29), no increase in FHR was triggered (Figures 5a–e).

Indeed, this activation is only possible when the SNS is properly developed. Our results provide evidence that this umbilico-neuro-cardiac effect, similar to the cardioprotective effect of remote preconditioning of trauma (RPCT). Discovered in 2009 by Jones et al. (29), RPCT showed that peripheral neural activation can provide cardioprotection in injured mice. This involves C-fiber stimulation at the umbilical level, triggering a dorsal root reflex (T9–T10 in mice) that activates cardiac sympathetic fibers(29). This stimulus may activate the same dorsal root reflex, corresponding to the T13–T14 spinal segments in sheep (44). One year later, researchers reported the presence of the somato-cardiac sympathetic C-reflex (45, 46). More recently, Wacker et al. (47) described a bone-neuro-heart reflex, where mechanical bone loading modulates heart rate via autonomic pathways in mice.

Building on these models, we propose that umbilical cord traction may trigger an umbilico-neuro-cardiac reflex. We hypothesize that this umbilico-neuro-cardiac reflex is triggered in two possible ways: first, by the stimulation of afferent C-fiber units; and second by the antidromic activation of efferent sympathetic C-fibers around the intrafetal portion of the umbilical cord (29, 48–50) caused by mechanical stimuli. Supporting this, TRPV1—the main C-fiber pain receptor—also modulates baseline sympathetic activity (51).

Thus, the results of the present study show that an abnormal increase in FHR can be ascribed to a mechanical and/or nociceptive stimulus (52, 53) that activates the SNS and triggers a cardiac response in the fetal lamb, which is equivalent to a similar feature in the human fetus.

The EXTEND system provides physiologic support and enables brain and cardiac development in fetal lambs (15, 16). In an earlier study (16), 105–111-day GA lambs were used to target pulmonary developmental equivalence (canalicular stage) to extreme preterm humans at 23–25 weeks of gestation. More recent studies have focused on investigating the EXTEND system’s ability to support 90–95-day GA lamb fetuses that more closely mimic the size and neurodevelopmental status of extremely premature human infants (15, 54).

The present study aimed to analyze the possible association between an increase in FHR and cord stretching, in ANS-mature vs. ANS-immature fetal lambs, as a potential explanation for clinical FHR events in the human fetus observed by CTG. While autonomic control of cardiac and vascular activity is developed in instrumented lambs at 110–111 days’ GA (17, 19) and in humans after 32 weeks’ GA (14), corresponding to a moderate-to-late preterm pregnancy, younger lambs have higher systemic vascular resistance and less autoregulatory capacity, leading to preferential shunting of cardiac output in the EXTEND system. The seminal 1975 study on instrumented fetal lambs demonstrating autonomic nervous system (ANS) responsiveness after 110 days of gestational age (GA) (17), showed a mature ganglionic response to various stimuli and effective vagal activity control, findings that were later confirmed by subsequent research (18, 19). This can be compared with human pregnancies at 32 weeks’ GA (14), in which ganglionic activity and the cerebral cortex resemble those in adults.

As stated above, modern CTG interpretation primarily uses a cardiovascular approach; however, based on the results of our experiments, this approach should be modified to consider this specific activity of the SNS. In particular, we should refocus on the interpretation of significant FHR increases, abnormal accelerations, or tachycardia as fetal responses to mechanical and nociceptive stimuli triggered by cord traction during or outside of labor in moderate to late preterm and term pregnancies. Indeed, by introducing this concept, it is possible to provide different interpretations for abnormal FHR increases both inside and outside of labor.

In particular, we should revisit the meaning of the so-called double mountain peak sign observed during labor, namely the presence of FHR accelerations whose peak coincides with the peak of the ongoing uterine contractions (21, 55). This sign is usually considered an erroneous monitoring of the maternal heart rate (MHR) mistaken for the FHR (21, 55, 56); however, according to our results, this should be attributed to mechanical activation of the umbilico-neuro-cardiac reflex due to traction of the umbilical cord. Thus, the double mountain peak sign on the CTG trace should be considered as an abnormal feature in labor but not because of an erroneous monitoring; rather, it is due to specific activation of the SNS (29) following an umbilical cord traction during a contraction. Therefore, a previous study showed that accelerations coinciding with uterine contractions occur on the CTG trace during active maternal pushing in 12% of cases (57), while, if internal monitoring using a fetal scalp electrode is employed, only 4% of the CTG traces show accelerations coinciding with uterine contractions during the second stage of labor (57). Instead of proving the contrary, this well-conducted study stated that at least 4% of the current fetal accelerations during labor cannot be explained by erroneous maternal monitoring or fetomaternal concurrence. Additionally, the authors clearly demonstrated that, although MHR could increase above FHR during active pushing, the two traces did not completely overlap (57). Furthermore, no study has shown a contemporaneous recording of maternal heart rate during acceleration in the same CTG trace (2).

Indeed, studies on human CTG patterns that revise the current literature, and prospective clinical trials could better address the issue of the SNS contribution to fetal myocardial activity. A potential limitation of the present study is its small sample size owing to the resource-intensive nature of maintaining fetal lambs on EXTEND. However, we perfectly matched the results of the power sample size calculations reported in statistics section. Additionally, although our results in the lamb model were consistent, the circulatory reaction and autoregulatory capacity of the human fetus should be investigated further.

## 5 Conclusion

Tachycardia in this experimental model on the EXTEND System appears to be specifically dependent on β-adrenergic pathways (58). Both cord traction and periumbilical capsaicin increased FHR and circulating norepinephrine, whereas propranolol abolished these responses. This is consistent with prior evidence that neuro-cardiac reflexes are mediated through sympathetic activation of β-adrenergic signaling. Our results suggest that permanent or transient tachycardia, whether occurring during or outside labor, when not accompanied by maternal tachycardia or laboratory abnormalities, may warrant reconsideration in a broader clinical context as a potential manifestation of mechanical or nociceptive stimulation, such as umbilical cord traction. Ultimately, unrecognized mechanical traction of the umbilical cord can lead to fetal demise or adverse perinatal outcomes, both during and outside of labor.

In summary, when assessing fetal wellbeing in moderate-to-late preterm and term pregnancies, more than only direct cardiovascular response to hypoxia/acidemia should be considered. As the current study on the EXTEND model clearly shows, different physiological responses to mechanical and nociceptive stimuli, such as umbilical cord stretching, should be considered as possible factors that can modify FHR in specific ways, primarily by triggering a significant FHR increase. Clinicians should be aware of this mechanism and promptly implement all necessary safety procedures to avoid possible adverse perinatal outcomes.

## Data Availability

The raw data supporting the conclusions of this article will be made available by the authors, without undue reservation.
