# Supplementary material for: Fetal heart and surroundings: umbilical cord traction triggers sympathetic heart rate surge in fetal lambs in the artificial womb
Source: Front Med (Lausanne). 2025 Nov 4;12:1667607. doi: 10.3389/fmed.2025.1667607 (PMC12696492; doi:10.3389/fmed.2025.1667607)
Supplement: Supplementary file 1 [file Table_1.DOCX]

|  | **Animal ID** | **Sex** | **Age at cannulation**  **(days)** | **Weight at cannulation**  **(kg)** | **Total days on the EXTEND support**  **(days)** | **Cause of the termination of the animal** |
| --- | --- | --- | --- | --- | --- | --- |
| 1 | 7780L1 | F | 93 | 0,95 | 16 | Suspected infection/ Not resolving metabolic acidosis |
| 2 | 2995L1 | F | 92 | 0,99 | 24 | Non resolving metabolic acidosis |
| 3 | 7954L1 | F | 91 | 0,86 | 22 | Non resolving metabolic acidosis |

**Supplementary Table 1. Characteristics of the animals included in the study.**
